# Public health implications of changing patterns of recruitment into the South African mining industry, 1973–2012: a database analysis

**DOI:** 10.1186/s12889-017-4640-x

**Published:** 2017-08-03

**Authors:** Rodney Ehrlich, Alex Montgomery, Paula Akugizibwe, Gregg Gonsalves

**Affiliations:** 1 Centre for Environmental and Occupational Health Research, School of Public Health and Family Medicine, University of Cape Town, Observatory, Cape Town, 7925 South Africa; 2 Data First, University of Cape Town, Cape Town, South Africa; 3 Global Health Justice Partnership, Yale Law School & Yale School of Public Health, New Haven, Connecticut USA

**Keywords:** Mining, Migrant labour, Gold, Tuberculosis, Silicosis, Southern Africa

## Abstract

**Background:**

The triple epidemic of silicosis, tuberculosis and HIV infection among migrant miners from South Africa and neighbouring countries who have worked in the South African mining industry is currently the target of regional and international control efforts. These initiatives are hampered by a lack of information on this population.

**Methods:**

This study analysed the major South African mining recruitment database for the period 1973 to 2012 by calendar intervals and demographic and occupational characteristics. Changes in area of recruitment were mapped using a geographic information system.

**Results:**

The database contained over 10 million contracts, reducible to 1.64 million individuals. Major trends relevant to health projection were a decline in gold mining employment, the major source of silicosis; increasing recruitment of female miners; and shifts in recruitment from foreign to South African miners, from the Eastern to the Northwestern parts of South Africa, and from company employees to contractors.

**Conclusions:**

These changes portend further externalisation of the burden of mining lung disease to home communities, as miners, particularly from the gold sector, leave the industry. The implications for health, surveillance and health services of the growing number of miners hired as contractors need further research, as does the health experience of female miners. Overall, the information in this report can be used for projection of disease burden and direction of compensation, screening and treatment services for the ex-miner population throughout Southern Africa.

**Electronic supplementary material:**

The online version of this article (doi:10.1186/s12889-017-4640-x) contains supplementary material, which is available to authorized users.

## Background

The South African mining industry has been built on migrant labour from the rural areas of South Africa and neighbouring countries, a system which although much reduced in size persists today. The numbers as well as geographical origin of these miners have changed over time in response to the changing needs of industry and to political and economic shifts in the region [1–4].

Prolonged or high exposure of miners to respirable silica dust released in the process of mining gold (and to a lesser extent coal) is the cause of silicosis, which now typically takes the form of a slowly developing lung fibrosis. Pulmonary tuberculosis is a frequent and potentially disabling complication of silica dust exposure and silicosis in this migrant population, as well as being the product of high rates of infection in congregate settings on the mines. Population tuberculosis risk is further elevated by a prevalence of HIV infection of the order of 25% [5, 6] and a synergistic effect of HIV infection and silicosis on such risk [7]. Consistent with these causal associations, studies of gold miners over the past two decades have confirmed high prevalences of silicosis of 13% to 25% in long service miners [8, 9] and high annual tuberculosis disease rates of 2500 per 100,000 and above [10]. Owing to the circular migration patterns of this workforce, high rates of tuberculosis among miners have translated into high tuberculosis rates in their home communities [11–13]. A similar association with migrant status has been found for HIV infection [14].

While employed miners on the larger mines have access to regular screening for tuberculosis and silicosis and to medical care, upon leaving the industry most of these ex-miners are lost to follow-up. However, their increased risk of tuberculosis due to silicosis and cumulative silica lung load is life-long [15], while silicosis itself may appear or progress after leaving employment [16]. Given the poorly developed health systems in the labour sending areas of Southern Africa, high rates of undiagnosed or incompletely treated tuberculosis are predictable, accompanied by poor access to the statutory compensation system for mining lung disease [6, 17–19].

This triple epidemic of silicosis, tuberculosis and HIV infection among miners drawn from the rural areas of South Africa and neighbouring countries, particularly Lesotho, Mozambique, Swaziland and Botswana, has belatedly become a target for regionally directed international action. In 2012 the Ministers of Health of the Southern African Development Community (SADC) committed their countries to reducing the burden of mining related lung disease [20]. Following a World Bank economic analysis of the benefits of treating and controlling tuberculosis among miners [21], the Global Fund to Fight HIV, Tuberculosis and Malaria committed ZAR 500 million (US$ 36 million) to the screening and treatment for tuberculosis of 500,000 miners, ex-miners and residents of peri-mining communities [22].

These initiatives have exposed the dearth of information on former miners – their whereabouts, occupational and other risk factors, health status, and changes in these phenomena over time. For example, while country or province of origin data on migrant miners have been reported [3, 13, 21, 23], these data have not been disaggregated to district level. The first objective of this study was thus to make use of a large recruitment database going back four decades to provide a current and historical description of the population of mainly migrant miners and ex-miners from the South African mining industry. The focus was on the large commodity sectors of platinum group metals (hereafter “platinum”), coal, and particularly gold mining as the highest risk industry for lung disease. A second objective was to draw out implications for the health status of this population with specific reference to silicosis and tuberculosis.

## Methods

### The TEBA database

An electronic database of contracts of miners recruited into the industry between 1973 and 28 October, 2013, was obtained from TEBA, an organisation that in various forms has been the major recruiting agency for the mines for since 1901 [2]. In 1977 its precursor organisation, the Witwatersrand Native Labour Association (WNLA), merged with the Native Recruiting Corporation (NRC) to form The Employment Bureau of Africa (TEBA). Owned for most of this period by the South Africa mining industry association, the Chamber of Mines, it became an independent private company, TEBA Ltd. (“TEBA” in this report) in 2005.

Personal communication from members of TEBA management constituted the source of information about the database in this and other sections. TEBA has a recruitment contract archive of 3 million person records. These records were held in hard copy until the 1970s. Digitisation of the archive commenced in the 1970s and electronic capture of the contracts of first time entrants to the industry was complete only for those entering from 1983 onwards, the year in which the mainframe computer was set up.

TEBA predominantly recruited for the gold mining industry, with a much smaller role in platinum and coal. From the late 1980s, there was a switch away from TEBA recruiting channels as South African nationality workers began to apply directly to the mines or through their union for employment. These contracts were not entered into the TEBA archive. However, TEBA continued to record all foreign nationality workers, who had to meet immigration requirements.

In response, TEBA shifted the emphasis of its operations by the 2000s from recruitment to processing, by opening offices on the mines and taking over the first step in the process of engagement of all employees. This greatly increased the numbers entered into the TEBA record system, including contractors (defined below) and non-migrant supervisory level (mainly white) miners. The companies Amplats (platinum) and Amcoal also transferred their labour registration process to TEBA over time.

### Analysis of the database

The database was converted by the researchers from one based on contracts [*N* = 10,327,396] to one based on individual miners [*N* = 1,644,264]. The process of organising, reducing and cleaning the original datafiles is described Additional file 1: Supplementary note 1. The variables available from the database are set out in Table 1.

**Table 1:** Variables from the TEBA database used in this study

Variable on database	Comment or further description	Derived Variables
Unique key	TEBA randomly assigned no. (Not personal ID number).	
Date of birth		Age
Gender		
Racial ascription	Four category classification formalised under apartheid, and in continuing usage for some official purposes – Asian, black, coloured, white.	
Origin	Country and district/region/province of office at which recruited or registered for a given contract.	Origin at first contract on the database
Contract start date		Number of contracts. Start date of first contract. Start dates of returning contract.
Contract end date		End date of first contract. End dates of returning contract.
Contract duration (days)	Difference between contract end date and contract start date.	Cumulative service across all contracts.
Contract duration (months)	Days or weeks rounded up or down to the nearest whole number of months, e.g. 35 days = 1 month.	Cumulative service across all contracts.
Commodity sector	Gold, platinum group metals, coal, “other” (antimony, asbestos, chrome, copper, diamond, granite, iron, lead, lime, tin, vanadium)^a^_._	Commodity sector of longest cumulative service for an individual.
Occupational risk category	Underground, “surface risk”, “surface non-risk”. Surface definition based on exposure to hazardous airborne agents.	Occupational risk category of longest cumulative service for an individual
Employment status	Mine employee versus “contractor”. See text for further definition.	Exclusively mine employee versus contractor “ever” on TEBA database

^a^Includes unassigned employees recorded as “Medical”, “Security”, “Training” and “Sundry”

With regard to employment status, although all miners have contracts, “contractor” is the term used on the TEBA database for miners who are employed by a subcontracting company or labour broker (“outsourcing” or “subcontracting”) rather than by the mining company itself, distinguishing them from “mine employees”. The database also has a “risk work” classification based on occupational respiratory hazards, mainly silica. All underground work and certain jobs on the surface (e.g. electricians, fitters, winding engine drivers) are categorised as risk work.

A total of 21% of miners had worked in more than one commodity sector and 36% in both surface and underground jobs. Where a unique assignment was needed for summary purposes, miners were assigned to the category in which they had accumulated the longest service. We elected not to use this method for employment status because of uncertainty about how many of the contractors registered on the TEBA database in the 2000s (when contracting became common) had unrecorded previous mine employee service. Employment status was thus dichotomised as exclusive mine employee only vs. “contractor ever”.

In order to assess the representativeness of the TEBA database with regard to industry employment, annual employment data were sought from sources other than TEBA. These sources and the comparisons made are described in Additional file 1: Supplementary note 2 and Additional file 2: Figure S1.

Frequencies, medians and ranges were calculated for variables of interest (listed in Table 1). Five-year intervals were used for comparison of changes over time. For the analysis of annualised cumulative service and secular trends, the database was truncated at 31 December, 2012.

### Geographic information system (GIS) mapping of new recruits

For purposes of mapping country, province and district of origin, only the first contract of each miners was used. Because it was not possible to determine from the database whether contracts recorded after 1973 were new or returning, the numbers mapped as new entrants are most reliable from 1983 onwards (on the assumption of a low probability of a miner being on a returning contract with no evidence of contracts in the previous decade).

The GIS software package ArcGIS 10.1 by ESRI was used. The mapping was disaggregated at the district level, and included miners from South Africa and neighbouring countries. During the geocoding step, district names associated with each miner (as recorded at the point at which they were recruited – not necessarily resident - for the first time) were assigned a latitude-longitude coordinate relating to their geographic location on the earth’s surface.

These coordinates were then integrated into a map interface, creating a spatial data layer of all miners recruited for each year since 1973. This provided a visual indication of the distribution of recruitment over time, broken down into 5-year periods, and aggregated at the district (or regional or country level for countries for which district subdivisions were not available).

## Results

### Date of entry into the industry

Table 2 presents the distribution of first entry into employment and of total employment by 5 year intervals. The sharp increase from 1973 to 1977 to 1983–1987 can be attributed largely to incomplete electronic registration of miners on the TEBA database in the decade 1973–1982. This is apparent from Additional file 2: Figure S1 which compares the TEBA figures to those published by the Chamber of Mines over that period. However, the latter still show a substantial real increase in employment over this early period.

**Table 2:** First entry of miners^a^ into TEBA database, and total employment by 5-year period, 1973–2013

Year of first entry	New entry^a^		Active in Period^b, c^	
	*n*	%	*n*	%
pre 1973	104,684	6.37	104,684	6.37
1973–1977	79,282	4.82	173,260	10.54
1978–1982	120,827	7.35	292,772	17.81
1983–1987	542,609	33.00	840,736	51.13
1988–1992	210,898	12.83	873,531	53.13
1993–1997	129,055	7.85	783,842	47.67
1998–2002	92,391	5.62	500,252	30.42
2003–2007	178,054	10.83	524,646	31.91
2008–2012	167,708	10.20	569,813	34.65
2013^d^	18,756	1.14	177,629	10.80
TOTAL	1,644,264	100.00	–	–

^a^Individual miners counted only once

^b^An individual miner cannot be counted more than once in a given period, but may be counted in more than one period

^c^Number and proportion of total number of miners who worked over the period 1973–2012 who were employed in that 5-year period

^d^Until 28 October, 2013

There was a sharp fall in new entry TEBA contracts after 1987, preceding by a decade a fall in total employment numbers (i.e. new and returning) from 1998. The number of new recruits recorded by TEBA increased somewhat from 2003.

### Person and employment characteristics

The predominance of gold mining, males, black miners and underground work in Table 3 reflects TEBA’s recruitment focus described earlier. (Additional file 3: Table S1 collapses the period data to provide the totals for the whole period.).

**Table 3:** Proportions (%) of miners^a^ in employment by 5-year period and demographic and occupational characteristics, 1973–2012

	1973–1977	1978–1982	1983–1987	1988–1992	1993–1997	1998–2002	2003–2007	2008–2012
Total	173,260	292,772	840,736	873,531	783,842	500,252	524,646	569,813
Country of origin								
South Africa	46.34	57.29	66	67.99	67.55	58.22	67.95	75.45
Other SADC	53.66	42.71	34	32.01	32.45	41.78	32.05	24.52
Racial ascription								
Black	96.85	95.83	97.17	93.65	91.01	90.5	91.04	90.72
White	3.09	4.11	2.77	6.22	8.8	9.14	8.44	8.58
Other categories	0.05	0.07	0.04	0.13	0.18	0.36	0.53	0.61
Gender								
Male	99.82	99.67	99.69	99.03	98.36	98.31	96.33	93.49
Female	0.18	0.33	0.31	0.97	1.64	1.69	3.67	6.51
Commodity^b^								
Gold	91.65	90.22	86.24	80.95	78.27	74.27	60.4	52.51
Platinum	6.45	7.75	11.2	11.47	11.42	14.61	25.28	32.32
Coal	0.77	0.92	1.04	4.22	4.23	3.07	2.96	2.37
Other	0.80	0.71	0.76	1.59	2.51	2.24	2.87	3.70
Unknown	0.33	0.39	0.77	1.77	3.57	5.8	8.5	9.11
Occupational risk category^b^								
Surface non-risk	8.31	9.76	16.99	10.59	9.09	12.89	11.45	9.02
Surface risk	0	0.01	0.01	0.02	0.06	0.29	2.15	8.6
Underground	91.69	90.23	82.99	89.39	90.85	86.82	86.4	82.38
Employment status								
Mine employee only	91.86	91.36	91.91	88.5	80.89	64.2	49.96	42.68
Ever contractor	8.14	8.64	8.09	11.5	19.11	35.8	50.04	57.32

^a^An individual miner cannot be counted more than once in a given period, but may be counted in more than one period

^b^Category where the longest service was accumulated over a full career if miner worked in more than one category

The most prominent time trends are the rise and then fall in aggregate employment (Table 3 and Additional file 2 Figure S1), decline in recruitment from neighbouring countries and growth in platinum, “contractor” and female recruitment. A total of just over 50,000 women appear on the database. Off a low base (< 2%), the relative proportion of female employment doubled in the period 2003–2007 and again in 2008–2012, reaching 6.5%.

Restriction of the above analysis to miners who had any contract days in the gold sector irrespective of where their longest service was (Additional file 3: Table S2) revealed similar trends. The decline from the late 1980s in the numbers with gold mining service is striking, from a cumulative total of 746,076 in the period 1983–1987 to 288,412 in the period 2008–2012 (Additional file 3 Table S2). This decline is confirmed by the external annualised data, which show a decline in annual gold mining employment from 553,449 in 1988, the year of peak employment, to 142,201 in 2012 (Additional file 2: Figure S1).

Overall, 453,487 individuals (27.9% of the total) were recorded as contractors on the TEBA database at some stage during their career (Additional file 3: Table S3). This group were more likely than mine employees to be female, South African nationality, white, in platinum or of unknown sector, and surface risk employees.

Table 4 indicates that median age at first entry into the industry was 25 years, the median length of contract 1 year and median cumulative service 6.3 years. Of the 1,223,152 miners recorded in the gold sector between 1973 and 2012, 221,054 (18.07%) had over 15 years of cumulative service and 119,931 (9.81%) over 20 years (not shown in table). The median cumulative service length in the gold sector (6.5 years) was greater than in platinum (3 years), coal (4.7 years) or other sectors (1 year).

**Table 4:** Age and employment characteristics of miners recorded on the TEBA database, 1973–2012

Characteristic	Median	Mean	Interquartile range	1st- 99th percentile	N^a^
Age at entry (yrs)	25	27.9	22–32	17–56	1,625,053
No. of contracts	4	6.3	1–9	1–26	1,625,053
Length of first contract (days)	365	669	350–429	8–5917	1,624,372
Length of returning contract (days)	365	448	357–392	8–3249	8,676,738
Cumulative employment – days (yrs)	2291 (6.3)	3062 (8.4)	732–4412 (2.0–12.1)	8 - 11,674 (0.02–40.0)	1,624,981
Average cumulative service by commodity – days (yrs)^b^					
Gold	2359 (6.5)	3093 (8.4)	755 to 4450 (2.1–12.2)	8 - 11,787 (0.02–32.3)	1,223,152
Platinum	1095 (3)	1787 (4.9)	367–2526 (1.0–6.9)	8–9013 (0.02–24.7)	392,806
Coal	1720 (4.7)	2441 (6.7)	366 to 3205 (1.0–8.8)	1 to 10,578 (0–29.0)	60,833
Other	374 (1.0)	838 (2.3)	365–911 (1.0–2.5)	0–5989 (0.02–16.4)	178,426

^a^Variation in totals due to exclusion of contracts with erroneous data, mainly termination date occurring before registration date. (Additional file 1 Supplementary note 1)

^b^All service days included here, irrespective of whether the miner worked in one or multiple sectors

### Place of recruitment

#### Province

Table 5 demonstrates that until the middle 2000s, within South Africa, the Eastern Cape was the province of predominant recruitment. It has recently been matched by steadily increasing recruitment in the North West province, where most of the platinum mining activity is concentrated as well as some gold mining. Third in ranking is Gauteng province, where gold mining predominates.

**Table 5:** Proportions (%) of miners^a^ in employment by 5-year period and province or country of origin, 1973–2012

	1973–1977	1978–1982	1983–1987	1988–1992	1993–1997	1998–2002	2003–2007	2008–2012
Total	173,260	292,772	840,736	873,531	783,842	500,252	524,646	569,813
Province of South Africa								
Eastern Cape	26.07	30.86	29.36	23.54	22.44	18.5	19.83	20.2
Northwest	5.52	7.22	10.08	10.41	9.63	9.48	15.29	19.62
Gauteng	4.44	5.09	5.27	9.69	13.29	13.57	13.62	13.73
Limpopo	2.38	3.17	4.12	5.25	4.53	3.1	4.47	6.07
Free State	4.45	5.29	8.12	8.56	8.78	7.81	8.35	8.53
KZN	2.99	4.97	7.97	8.69	7.41	4.49	3.8	3.81
Mpumalanga	0.34	0.52	0.82	1.6	1.24	1.01	2	2.52
Western Cape	0.01	0.01	0.01	0.02	0.02	0.02	0.05	0.09
Northern Cape	0.12	0.16	0.26	0.24	0.21	0.25	0.55	0.9
Other country								
Lesotho	26.89	21.76	15.17	15.15	15.65	18.71	14.35	11.13
Mozambique	16.57	11.37	9.34	9.14	12.19	17.81	14.22	10.82
Swaziland	2.42	2.52	2.78	2.78	2.6	3.01	2.22	1.65
Botswana	5.09	4.33	3.09	2.55	2	2.23	1.24	0.84
Malawi	2.54	2.51	3.53	2.39	0.01	0.01	0	0

^a^An individual miner cannot be counted more than once in a given period, but may be counted in more than one period

To provide a picture of relative recruitment over the whole period 1973–2013, Additional file 3:Table S4 presents the aggregate figures for area of origin restricted to first time entrants. The overall ranking remains unchanged.

Of the surrounding countries, Lesotho has historically provided the largest proportion of miners on the database (12.9%) (Additional file 3:Table S4), although declining from 26.8% in 1973–1978 to 11.1% in 2008–2012 (Table 5). Recruitment from Mozambique has ranked second (9.11%), but has followed a different time trend, declining initially but recovering in the 2000s and matching that of Lesotho.

#### District

**Fig. 1:**
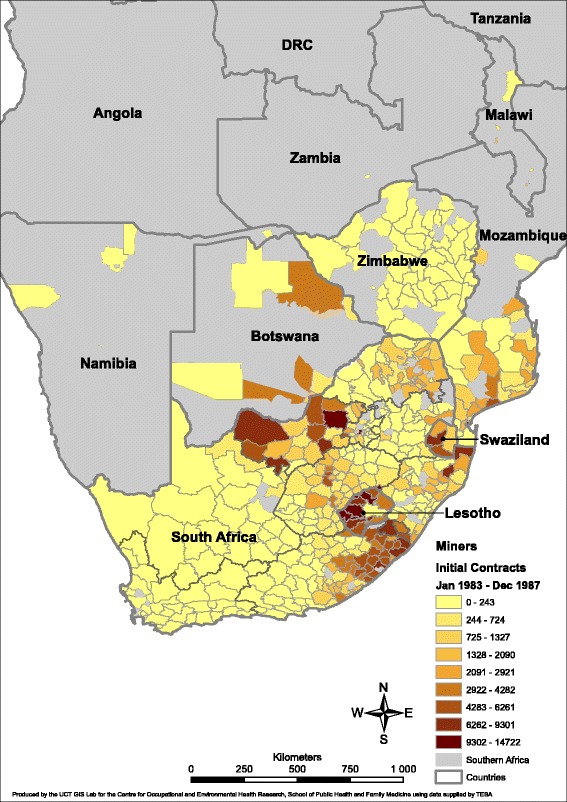
Distribution of recruits to the South African mining industry at first contract, by district, 1973–1978. *DRC*: Democratic Republic of Congo

**Fig. 2:**
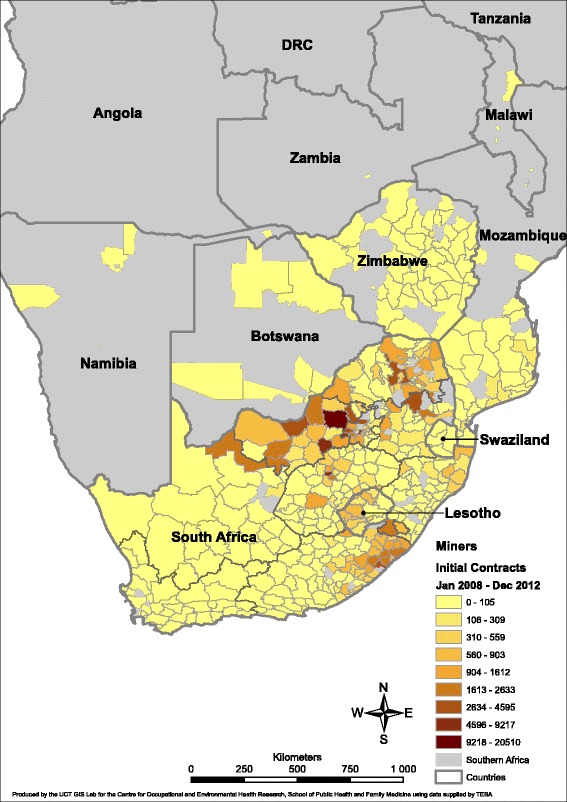
Distribution of recruits to the South African mining industry at first contract, by district, 2008–2012. *DRC*: Democratic Republic of Congo

Figs. 1 and 2 demonstrate the GIS mapping of mine recruitment from districts of South Africa and surrounding countries. Because of the wide range in total numbers across time periods, the numeric range associated with any given colour/shade is not the same in the two maps. Any given colour or shade should thus be read as a *relative position* when looking at different periods rather than representing the same numeric range in both maps.

Figure 1 illustrates the distribution in the period of peak recruitment 1983–1987. The South African districts with approximately 4300 to 15,000 recruits (i.e. the range across the top three recruitment strata) are heavily concentrated in the eastern half of the Eastern Cape (Transkei region), the Northwest province (previously Western Transvaal/ Bophuthatswana) and Northern Kwazulu-Natal (previously Natal). The surrounding countries with heavy district recruitment are Lesotho, Swaziland, Botswana and (southern) Mozambique. The few “stand alone” South African districts are the mining or urban recruitment areas of Johannesburg in Gauteng province, and Witsieshoek (a recruitment area) and Welkom (a mining area) in the Free State province.

Figure 2 enables comparison of the above with the relative district distribution for the latest available period 2008 to 2012. The top three recruitment strata cover approximately 2500 to 21,000 recruits in this figure, but lower on average than in 1983–1988 in keeping with lower recruitment numbers overall. Visually, it is evident that there is a shift westward and to some extent northward, of the areas of highest recruitment. This reflects the shift away from surrounding countries and from Kwazulu-Natal and the Eastern Cape. The Northwest province remains prominent, dominated by Rustenberg and Klerksdorp, with a number of districts in Gauteng now standing out, as well as individual districts in the provinces of Free State (Welkom), Mpumalanga (Lydenberg) and Limpopo (Pietersburg). Additional file 4: Figure S2, which maps the whole period 1973 to 2012, shows a similar picture to that of Fig. 1, reflecting the numeric weight of the higher numbers recruited in the earlier period.

## Discussion

This report is to our knowledge the first published attempt to map the recruitment origin of this workforce, and particularly the black migrant workforce, to the district level, as well providing a detailed description of occupational and demographic characteristics. The main trends are a decline in gold mining employment and rise in platinum employment, a decline in foreign recruitment, a shift in point of recruitment northwards and westwards and an increase in the number of female miners. There was also a substantial increase in the employment of contractors. These findings and their health implications are discussed below.

### Commodity sector

During the first three decades covered in this analysis, gold mining employment dominated the TEBA database. Striking feature features are the increase in mining employment from the mid-1970s through the 1980s (Additional file 4: Figure S2), a time of a rising gold price, and the decline in new recruits after 1988 at a time of retrenchment following a major miners’ strike in 1987. The finding that this decline was not matched by a corresponding fall in total employment may be partly due to re-engagement of many of the retrenched workers [24] with preferential re-employment of previously employed miners. However, a long-term fall in total mining employment becomes apparent from the late 1990s, driven by a decline in gold mining employment (Table 3, Additional file 3: Table S2 and Additional file 2: Figure S1).

This decline has important implications for public health outside the formal employment sector. Gold mining has been the main generator of occupational lung disease in the form of silicosis and related tuberculosis [13]. As the ratio of ex-gold miners, including many with long service, to working gold miners rises, so does the “externalisation” of disease from the industry to community settings [9]. Continued shedding of jobs is forecast for the gold mining industry in response to economic and political difficulties.

Employment numbers in the platinum mining industry passed those of gold in 2006 during platinum’s phase of rapid expansion [25]. While platinum mining in South Africa carries a very low risk of silicosis, about a half of its miners have worked in other commodity sectors [26], thus carrying their risk factors, including lung silica load and silicosis from gold mining, into the platinum sector. However, the platinum industry is also currently in economic crisis owing to rising costs and a fall in demand for the metal, which is likely to further reduce the employment opportunities for former gold miners.

### Duration of exposure to silica dust

The available exposure variables most relevant to silicosis and related tuberculosis are time spent in underground or surface risk work, particularly on gold mines. The large majority of miners work underground or have mixed underground/surface exposure. While the median gold miner entered employment at 25 years of age for a service duration of 6.5 years, over the period covered by this database more than 220,000 gold miners accumulated service of 15 years or more. While a figure of 15–20 years is frequently used as a “threshold” for the appearance of radiological silicosis under conditions of dust exposure over the past half century, compensation data from the 1970s to 1990s suggest that as many as a half of black gold miners with silicosis claims had accumulated less the 15 years of service [27].

In addition, many miners have silicosis that is not detectable on chest x-ray [28]. Autopsy data of gold miners dying of non-natural causes (presumed to be unrelated to lung disease risk) show that 4% of black miners with less than 10 years of service and 13% with 10 to 14 years of service have autopsy evidence of silicosis in their lungs [29]. These two low duration categories make up the majority of gold miners coming to autopsy from non-natural causes. Thus even relatively short service ex-gold miners may remain at risk of later progression of silicosis to the radiological stage, although information on the extent of this “long latency appearance” of silicosis is lacking.

With regard to tuberculosis risk, there is evidence of a low silicosis threshold for the increase in tuberculosis risk, with an elevated relative risk observable in the lowest category of both autopsy silicosis (“negligible”) [15) and radiological silicosis [7] (“possible”, i.e. International Labour Organisation category 0/1) [7, 30].

These figures have implications for any public health programme designed to detect tuberculosis and silicosis in ex-miners. The first is that while the number of gold miners on the TEBA database still alive who have worked over the past four decades and who have accumulated 15 or more of service is unknown, on an estimate of 60% still living (out of a denominator of 221,054 long service miners), this group is of the order of 133,000 individuals. This is a minimum, as it excludes the living fraction of miners who bypassed the database. The second implication is that even among those with shorter service histories there is an elevated lifelong risk of tuberculosis due to accumulated silica load and sub-radiological silicosis. Taken together with a high prevalence of HIV infection and background tuberculosis risk in their home communities, this group is likely to have a high lifelong annual risk of active tuberculosis.

### Female miners

The rise in employment of women in the mining industry, particularly in risk work, is a recent phenomenon, linked to the Mining Charter of 2002, a negotiated code gazetted as a regulation in 2004, which set a target of 10% for participation by women in the industry [31]. As women accumulate exposure in dusty areas, they are likely to be subject to similar rates of disease as men in those areas. There has not to our knowledge been any study of mining related disease among women, neither silicosis nor tuberculosis, and such research will be needed.

### Place of recruitment

There is now a large literature on the oscillating migration of large numbers of miners recruited from surrounding countries and the rural Eastern Cape of South Africa, and on the profound socioeconomic impact of the migrant system on life in these regions [4, 23, 32–34]. The trend over the past four decades has been for labour from surrounding countries to be replaced by South African nationals, with the proportion of foreign nationals declining from over half of all miners employed in the 1973–1978 period to a quarter in 2008–2012. The decline in the previously large number of Malawian recruits dates from 1974, with some reinstatement thereafter until complete cessation in the late 1980s. Reasons for these changes are varied, with pressures originating both in labour sending countries such as Malawi and Mozambique, and in South Africa, with changes in immigration laws and political and economic pressures on the industry to recruit domestically [4, 23, 32, 34].

Within South Africa, the major shift of point of recruitment has been from the Eastern Cape to the Northwest province, particularly Rustenberg, and to other areas of mining or urban recruitment activity. This shift reflects the replacement of the gold by the platinum industry as the major employer of labour, the signing on by migrant miners directly at the mine rather than in their home district, and a policy of recruiting increasingly from surrounding rather than remote areas within South Africa.

Studies measuring the burden of silicosis and tuberculosis among former miners are sparse, limited almost entirely to former gold miners from specific districts in the Transkei [35] and Botswana [36] and to a cohort off laid off miners from Lesotho [6, 8]. There are none to our knowledge from Swaziland nor Mozambique. The change in the geographic and sectoral concentrations of miners described above calls for community studies in these areas, where miners with both platinum and gold mining history now live. The information from such studies would inform education, screening, treatment and compensation services in these areas.

### Contractor vs. mine employee status

The proportion of miners who had worked at least one contract as a contractor accelerated sharply as a proportion of those employed after 1998–2002, reaching over half of the total (and gold mining) workforce by 2008–2012. Contractors are a heterogeneous category, including miners hired via labour brokers, skilled workers employed primarily in surface work and those employed by established specialist companies contracted to undertake some stage of the mining process, such as shaft sinking [23]. Because of this heterogeneity, it is difficult to state general hypotheses about the implications of contractor status for health and well-being. These implications are likely to be adverse where contractor status entails precarious employment in the sense of lower earnings, fewer benefits, and less access to routine medical surveillance and medical services than company employees [23]. Whether contractor status translates into a higher risk of occupational lung disease is not known. This category needs to be disaggregated by precariousness of employment, occupational level and length of service as a contractor to answer this question.

### Limitations

The TEBA database is not a complete representation of mining employment. For the period 1973–1983, it is clear that the database numbers are an undercount (Additional file 2: Figure S1). However, there is no reason to believe that demographic and occupational characteristics were greatly distorted by the undercount, an assumption supported by the consistency of these proportions with those of the immediately succeeding period.

After 1983 TEBA figures exceed or are very close to those from the two other sources used (Additional file 2: Figure S1). It is likely that the database provides a complete picture of black, and therefore migrant, gold mining employment during the 1980s. Because workers from other countries, whether contractors or mine employees, had to go through TEBA registration channels, the database is also likely to be a near complete representation of foreign migrant labour. Despite the observation by a TEBA official that many recruits from its traditional South African recruitment population bypassed TEBA in the 1990s, Additional file 2: Figure S1 does not show an undercount compared to government figures, which suggests that the latter may also be an undercount. The bypass factor would have diminished after 2003 with the shift in the TEBA modus operandi to registration from recruitment. The increase in the proportions of white workers and surface jobs on the database is likely to be an artefact of this shift.

With respect to platinum and coal, the TEBA database representativeness was incomplete from the beginning of the period of interest because of competing recruitment channels. Registration of platinum miners increased substantially after 2003, with a persistent but narrowing shortfall compared to government employment figures (Additional file 1 Supplementary note 2). TEBA has always been a minority recruiter of coal workers, who in South Africa have a lower risk of pneumoconiosis than gold miners [37], although studies of the coal mining workforce remain scarce.

Finally, since miners with multiple contracts were assigned to area of registration at first contract, we could not identify miners who who signed up for their first contract or for later contracts at localities different from their home area. This size of this discrepancy is unknown but is likely to have increased over time for the reasons discussed earlier.

## Conclusions

The objective of this report was to provide a descriptive rather than an explanatory analysis of changing recruitment patterns in the South African mining industry, with a focus on their implications for the overlapping domains of occupational and public health.

All miners and ex-miners from the South African industry are by statute entitled to medical examinations and monetary compensation for occupational disease. While miners who are actively employed usually have access to medical services for these diseases, including treatment, once they leave the industry the responsibility for their health surveillance passes from the industry mainly to the state. This system of post-employment health surveillance works very poorly [18, 38, 39], and as a result many such ex-miners are lost to the health and compensation systems. For example, a survey of rural ex-miners found that 62% of those eligible had not been compensated, 35% partially compensated (i.e. not for disease progression) and only 2.5% fully compensated [35]. This barrier to access is not only an individual and public health concern but reflects the failure of a century of legislation resulting from miner struggles to secure compensation for these diseases. Amelioration is thus a matter of social justice.

The detection and treatment of tuberculosis among miners, former miners and their communities are currently the target of a huge international effort. A 2011 modelling study suggested that 760,000 new cases of tuberculosis in sub-Saharan Africa every year may be linked to mining activity across the subcontinent [12]. Control of the epidemic of tuberculosis, HIV and silicosis in miners and ex-miners in Southern Africa is therefore both an occupational health and a public health priority. This cannot be done without knowing where the miners or former miners and their families are.

Based on the region’s primary recruitment database, the study establishes an epidemiological and spatial framework that can be used for disease projection, costing and planning of programmes, including access to medical examinations and compensation, for a hard to reach and long neglected population. The findings should also assist actuarial costing of the compensation liability as part of current attempts to reform the compensation system.

## Additional files

Additional file 1: Supplementary note 1. Reduction and cleaning of database. **Supplementary note 2**: Comparison of TEBA figures with external sources. (DOCX 25 kb)

Additional file 2: Figure S1. Comparison of TEBA annual employment figures in the gold sector with figures from other sources, 1973–2012. Additional file 1 Supplementary note 2 for sources. *TEBA:* TEBA Ltd. *COM*: Chamber of Mines. (TIFF 36 kb)

Additional file 3: Table S1. Demographic and employment characteristics of mineworkers at first entry recorded on TEBA Database, 1973–2012 (*N* = 1,625,053). **Table S2.** Proportions (%) of mineworkers in the gold sector on TEBA database active during successive 5-year periods, by demographic and occupational characteristics, 1973–2012. **Table S3.** Demographic and occupational characteristics of exclusively mine employees versus those who were recorded as a contractor at some point on the TEBA database, 1973–2012. **Table S4.** Province or country of recruitment of mineworkers recorded on the TEBA database, by place at first recruitment, 1973–2012. (DOCX 35 kb)

Additional file 4: Figure S2. Distribution of recruits to the South African mining industry at first contract, by district, 1973–2012. (PDF 1370 kb)

## Abbreviations

GIS: Geographic information system
HIV: Human immunodeficiency virus
