# Supplementary material for: Public health implications of changing patterns of recruitment into the South African mining industry, 1973–2012: a database analysis
Source: BMC Public Health. 2017 Aug 3;18:93. doi: 10.1186/s12889-017-4640-x (PMC5543439; doi:10.1186/s12889-017-4640-x)
Supplement: Supplementary file 1 — Reduction and cleaning of database. Supplementary note 2: Comparison of TEBA figures with external sources. (DOCX 25 kb) [file 12889_2017_4640_MOESM1_ESM.docx]

**ADDITIONAL FILE 1**

**Supplementary note 1. Reduction and cleaning of database**

Organisation

The data were received from TEBA as a relational table in which each row entry represented a contract. Each miner was identified in the datafile by a unique key. A separate table with "contractor" status contracts was included alongside the original datafile of company employee contracts. These were combined to create a larger table which represented both kinds of employment status.

The larger dataset had information on registration dates and termination dates for each contract, the commodity in which the miner worked for that contract, the risk type (underground, surface risk or surface non-risk) of each contract, the point of origin of each miner for that contract, demographic variables, and some other information. More variables could be then derived using this information. For example, contract length was determined by subtracting start date from end date and adding one (to ensure that same day contracts counted as being one day long).

Reduction

A total of 10 327 396 first time and returning contracts were recorded on the TEBA database. These contracts were undertaken by 1 644 264 individual miners. Of these, 18 756 were recruited in 2013. A further 104 684 who commenced mine service before 1973 were included as returning miners but were excluded in any analysis of new entry contracts

Contracts ending prior to 1973 were excluded from the analysis, as were the less than 0.5% of the miners recruited from the non-African countries.

Linkage to individuals

After deriving numerous variables the data were collapsed into wider but shorter individual level files using the unique key identifier in the contract table.

Cleaning

Of the total number of contracts, 338 999 (3.4%) had no termination dates. As the majority of these contracts started close to the last database contract registration date of 28 October, 2013, an assumption was made that these were ongoing contracts. Accordingly, these were censored at the above date. A further 5 781 contracts (5 534 individuals) had termination dates predating the registration date. This was assumed to be an error and these contracts were omitted from calculations of cumulative service.

**Supplementary note 2. Comparison of TEBA figures with external sources**

Different agencies, both government and industry, collect, collate and publish annual mining employment figures in South Africa, often drawing on each other’s data. It is therefore difficult to find a set of figures across the whole period of interest, 1973-2012, which has not made use of TEBA employment figures at some stage.

Two sources have therefore been used. For the period 1973-1993, gold employment figures were obtained from the Chamber of Mines whose members are responsible for most gold mining employment in South Africa. These figures are not necessarily independent of TEBA, but served the purpose here of providing a benchmark for TEBA’s digitised gold employment figures in the run-in period 1973-1983. For the period 1994-2012, gold and platinum employment figures were obtained from Department of Minerals and Energy, later Department of Mineral Resources, source.

The gold employment figures are graphically depicted in Figure S1. This is the most relevant comparison for purposes of this report.

With regard to platinum employment, TEBA’s figures are lower than that of the government departments throughout the period 1994-2012. (See main text for explanation). There has been some narrowing of the TEBA “underregistration” gap - from approximately 70 000 in 2003 to 43 000 in 2011-2012.

References

1. Chamber of Mines of South Africa. Facts and Figures (up to 1992). Johannesburg: Chamber of Mines (Information Service: info@chamberofmines.org.za)

2. Department of Mineral Resources. Minerals Statistical Tables 1993 – 2014. 17th edition, with b2 Statistical tables (spreadsheets). Pretoria: Directorate: Mineral Economics, Bulletin B1/2015, November 2015. <http://www.dmr.gov.za/publications/mineral-economics.html> ("Statistics" tab).
