# Supplementary figures and images for: Public health implications of changing patterns of recruitment into the South African mining industry, 1973–2012: a database analysis
Source: BMC Public Health. 2017 Aug 3;18:93. doi: 10.1186/s12889-017-4640-x (PMC5543439; doi:10.1186/s12889-017-4640-x)

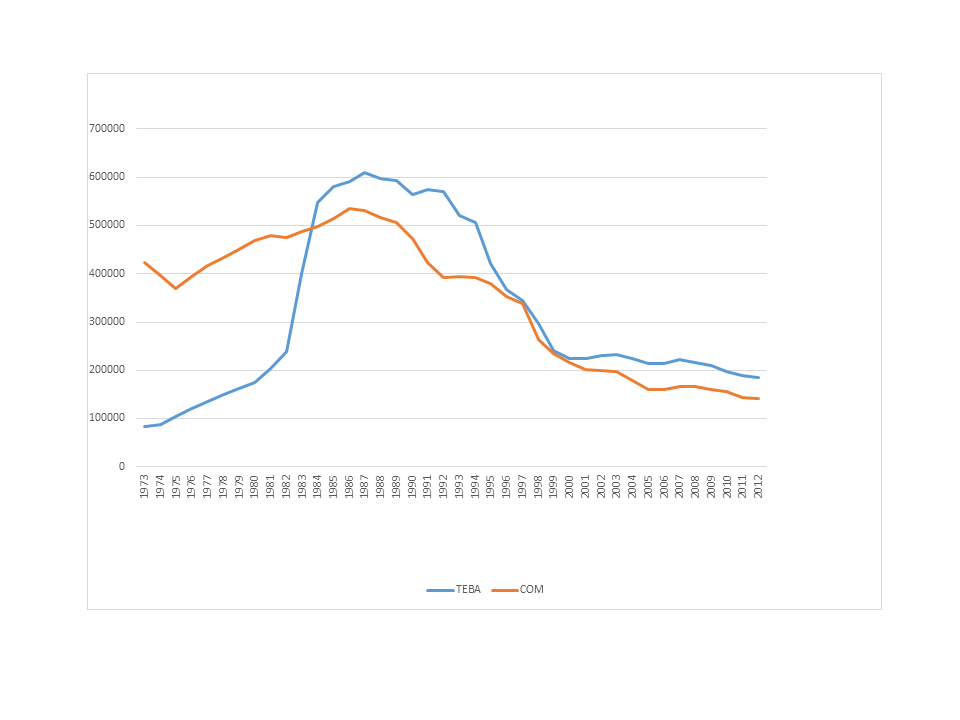

Supplement: Supplementary file 2 — Comparison of TEBA annual employment figures in the gold sector with figures from other sources, 1973–2012. Additional file 1 Supplementary note 2 for sources. TEBA: TEBA Ltd. COM: Chamber of Mines. (TIFF 36 kb) [file 12889_2017_4640_MOESM2_ESM.tif]

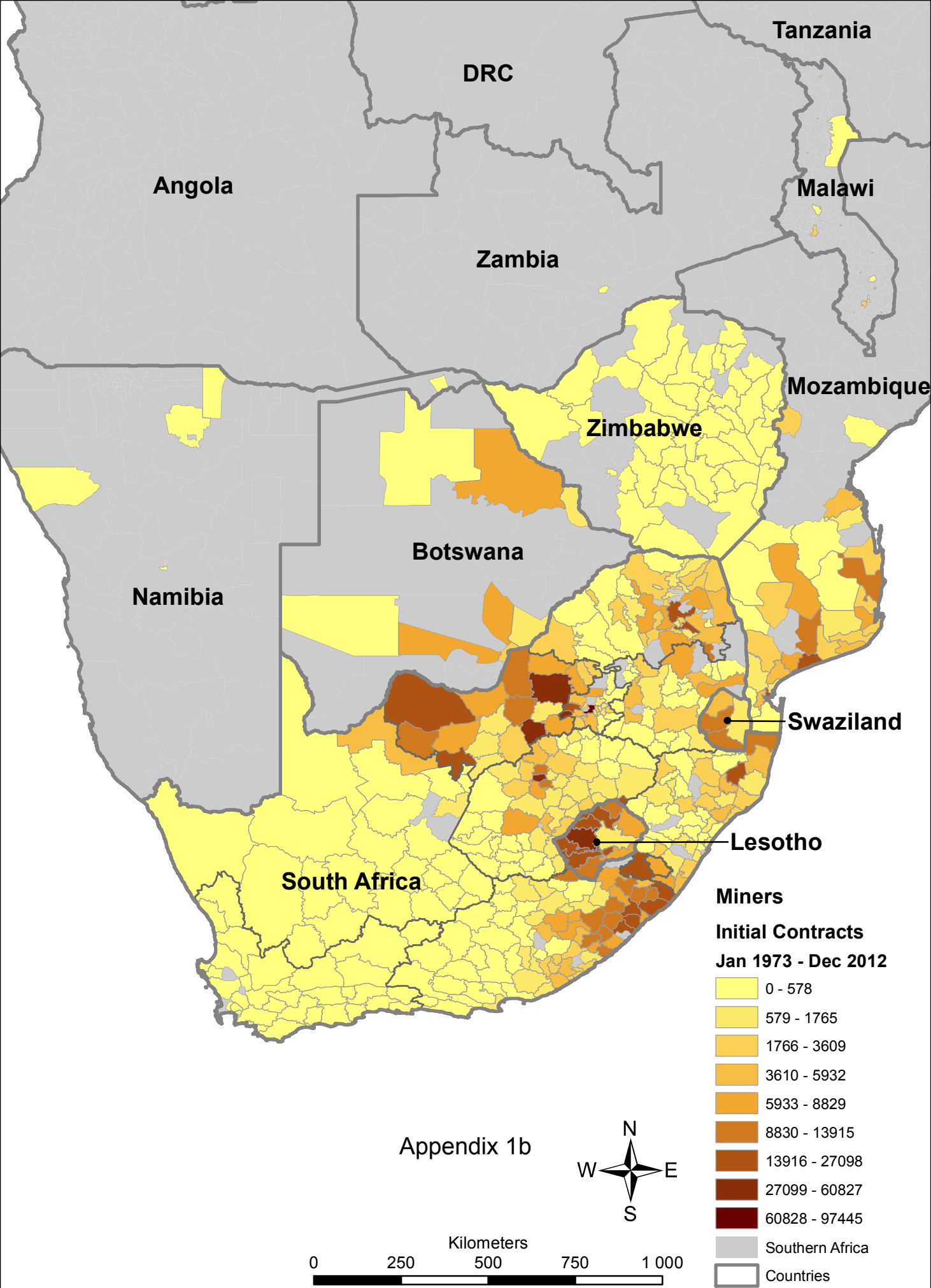

Supplement: Supplementary file 4 — Distribution of recruits to the South African mining industry at first contract, by district, 1973–2012. (PDF 1370 kb) [file 12889_2017_4640_MOESM4_ESM.pdf]
