# Supplementary material for: Public health implications of changing patterns of recruitment into the South African mining industry, 1973–2012: a database analysis
Source: BMC Public Health. 2017 Aug 3;18:93. doi: 10.1186/s12889-017-4640-x (PMC5543439; doi:10.1186/s12889-017-4640-x)
Supplement: Supplementary file 3 — Demographic and employment characteristics of mineworkers at first entry recorded on TEBA Database, 1973–2012 (N = 1,625,053). Table S2. Proportions (%) of mineworkers in the gold sector on TEBA database active during successive 5-year periods, by demographic and occupational characteristics, 1973–2012. Table S3. Demographic and occupational characteristics of exclusively mine employees versus those who were recorded as a contractor at some point on the TEBA database, 1973–2012. Table S4. Province or country of recruitment of mineworkers recorded on the TEBA database, by place at first recruitment, 1973–2012. (DOCX 35 kb) [file 12889_2017_4640_MOESM3_ESM.docx]

**ADDITIONAL FILE 3**

Table S1

Demographic and employment characteristics of miners at first entry recorded on TEBA Database, 1973-2012 (N = 1 625 053)^a^

| **Characteristic** | **Number** | **Proportion (%)** |
| --- | --- | --- |
|  |  |  |
| **Gender** |  |  |
| Male | 1 574 637 | 96.90 |
| Female | 50 416 | 3.10 |
| **Country of origin**^b^ |  |  |
| South Africa | 1 189 515 | 73.20 |
| Other SADC | 435 423 | 26.79 |
| Other Country | 115 | 0.01 |
| **Racial ascription** |  |  |
| Black | 1 503 206 | 92.50 |
| Coloured | 5 441 | 0.33 |
| Indian | 1 060 | 0.07 |
| Other | 342 | 0.02 |
| White | 115 004 | 7.08 |
| **Commodity^c^** |  |  |
| Gold | 1 136 067 | 69.91 |
| Platinum | 311 567 | 19.17 |
| Coal | 53 202 | 3.27 |
| Other | 41 442 | 2.55 |
| Unknown | 82 775 | 5.09 |
| **Occupational risk category^c^** |  |  |
| Surface | 239 739 | 14.75 |
| Surface Risk | 52 226 | 3.21 |
| Underground | 1 333 088 | 82.03 |
| **Employment status** |  |  |
| Mine employee only | 1171 566 | 72.09 |
| Contractor ever | 453 487 | 27.90 |

^a^ Discrepancy from original total due to missing data and exclusion of mineworkers recruited after 2012.

^b^ At entry.

^c^ Individual miners assigned to category of longest cumulative service.

Table S2

Proportions (%) of miners^a^ in the *gold sector*^b^ on TEBA database active during successive 5-year periods, by demographic and occupational characteristics, 1973-2012

|  |  |  |  |  |  |  |  |  |
| --- | --- | --- | --- | --- | --- | --- | --- | --- |
|  | **1973-1977** | **1978-1982** | **1983-1987** | **1988-1992** | **1993-1997** | **1998-2002** | **2003-2007** | **2008-2012** |
| Total (numbers) | 162 430 | 271 526 | 746 076 | 720 874 | 625 166 | 383 753 | 322 686 | 288 412 |
| **Country of origin** |  |  |  |  |  |  |  |  |
| South Africa | 45.58 | 56.44 | 64.25 | 65.18 | 66.32 | 57.76 | 63.68 | 72.45 |
| Other country | 54.42 | 43.56 | 35.75 | 34.82 | 33.68 | 42.24 | 36.32 | 27.55 |
| **Racial ascription** |  |  |  |  |  |  |  |  |
| Black | 97.13 | 96.26 | 97.46 | 94.53 | 91.92 | 91.35 | 91.56 | 90.76 |
| White | 2.81 | 3.68 | 2.49 | 5.35 | 7.90 | 8.30 | 7.92 | 8.49 |
| Other categories | 0.06 | 0.06 | 0.05 | 0.12 | 0.18 | 0.35 | 0.52 | 0.76 |
| **Gender** |  |  |  |  |  |  |  |  |
| Male | 99.84 | 99.72 | 99.72 | 99.23 | 98.64 | 98.47 | 96.86 | 94.02 |
| Female | 0.16 | 0.28 | 0.28 | 0.77 | 1.36 | 1.53 | 3.14 | 5.98 |
| **Occupational risk category**^c^ |  |  |  |  |  |  |  |  |
| Surface non-risk | 7.94 | 8.64 | 15.31 | 9.76 | 8.81 | 13.61 | 13.13 | 11.67 |
| Surface Risk | 0 | 0.01 | 0.01 | 0.02 | 0.05 | 0.24 | 1.34 | 6.39 |
| Underground | 92.05 | 91.35 | 84.68 | 90.22 | 91.14 | 86.16 | 85.53 | 81.94 |
| **Employment Category**^\^ |  |  |  |  |  |  |  |  |
| Mine employee only | 91.74 | 91.13 | 91.37 | 87.63 | 80.04 | 63.58 | 49.38 | 41.38 |
| Contractor ever | 8.26 | 8.87 | 8.63 | 12.37 | 19.96 | 36.42 | 50.62 | 58.62 |

^a^ An individual miner cannot be counted more than once in a given period, but may counted in more than one period.

^b^ Includes all miners who spent any contract days in the gold sector irrespective of whether they worked in other sectors as well.

^c^ Individuals assigned to category of longest cumulative service.

Table S3

Demographic and occupational characteristics of exclusively mine employees versus those who were recorded as a contractor at some point on the TEBA database, 1973-2012

|  | **Exclusively mine employee** | | **Contractor ever** | |  |
| --- | --- | --- | --- | --- | --- |
| **Characteristic** | **N** | **%** | **N** | **%** | **Total** |
| **Total** | 1 171 566 | 72.09% | 453 487 | 27.88% | 1 625 053 |
| **Gender** |  |  |  |  |  |
| Male | 1 140 078 | 97.31% | 434 559 | 95.83% | 1 574 637 |
| Female | 31 488 | 2.69% | 18 928 | 4.17% | 50 416 |
| **Country of origin** |  |  |  |  |  |
| South African | 830 767 | 70.91% | 358 748 | 79.11% | 1 189 515 |
| Other country | 340 799 | 29.09% | 94 739 | 20.89% | 435 538 |
| **Racial ascription** |  |  |  |  |  |
| Black | 1 098 586 | 93.77% | 404 620 | 87.14% | 1 503 206 |
| White | 70 711 | 6.04% | 44 293 | 11.22% | 115 004 |
| Other categories | 2 269 | 0.19% | 4 274 | 1.64% | 6 843 |
| **Commodity** ^a^ |  |  |  |  |  |
| Gold | 884 448 | 75.49% | 251 619 | 55.49% | 1 136 067 |
| Platinum | 195 585 | 16.69% | 115 982 | 25.58% | 311 567 |
| Coal | 50 212 | 4.29% | 2 990 | 0.66% | 53 202 |
| Other | 31 394 | 2.68% | 10 048 | 2.22% | 41 442 |
| Unknown | 9 927 | 0.85% | 72 848 | 16.06% | 82 775 |
| **Occupational risk category**^a^ |  |  |  |  |  |
| Surface | 188 476 | 16.09% | 51 263 | 11.30% | 239 739 |
| Surface risk | 6 423 | 0.55% | 45 803 | 10.10% | 52 226 |
| Underground | 976 667 | 83.36% | 356 421 | 78.60% | 1 333 088 |

^a^ Individual miners assigned to sector and occupation of longest cumulative service.

Table S4

Province or country of recruitment of miners recorded on the TEBA database, by origin at first recruitment, 1973-2012

| **Province of South Africa** | **Numbers** | **Percentage** |
| --- | --- | --- |
| Eastern Cape | 373 160 | 23.67 |
| Northwest | 231 438 | 13.75 |
| Gauteng | 200 826 | 11.26 |
| Limpopo | 92 248 | 5.18 |
| Free State | 139 687 | 8.37 |
| Kwazulu-Natal | 109 387 | 6.75 |
| Mpumalanga | 33 946 | 1.97 |
| Northern Cape | 7 994 | 0.48 |
| Western Cape | 829 | 0.04 |
| **Other country^a^** |  |  |
| Lesotho | 191 225 | 12.58 |
| Mozambique | 152 091 | 9.91 |
| Swaziland | 31 958 | 2.10 |
| Botswana | 29 224 | 1.95 |
| Malawi | 29 741 | 1.93 |
| Other Southern African | 1 184 | 0.03 |
| **TOTAL** | **1 624 938^a^** | **100** |

^a^ Excludes 157 from outside Southern Africa.
